# The long noncoding RNA *lncPARP1* contributes to progression of hepatocellular carcinoma through up-regulation of PARP1

**DOI:** 10.1042/BSR20180703

**Published:** 2018-06-21

**Authors:** Heqiang Qi, Yuyan Lu, Jie Lv, Huita Wu, Jing Lu, Changmao Zhang, Sheng Zhang, Qing Bao, Xiuming Zhang, Chengrong Xie, Zhenyu Yin

**Affiliations:** 1 Department of Hepatobiliary Surgery, Zhongshan Hospital, Xiamen University, Fujian Provincial Key Laboratory of Chronic Liver Disease and Hepatocellular Carcinoma, Xiamen, Fujian, P.R. China; 2 Department of Oncology, Zhongshan Hospital, Xiamen University, Xiamen, Fujian, P.R. China; 3 Faculty of Clinical Medicine, Fujian Medical University, Fuzhou, Fujian, P.R. China; 4 Department of Biomaterials, College of Materials, Xiamen University, Xiamen, Fujian, P.R. China

**Keywords:** growth, invasion, PARP1, prognosis

## Abstract

Hepatocellular carcinoma (HCC) accounts for a large proportion of cancer-associated mortality worldwide. The functional impact of long noncoding RNAs (lncRNAs) in human cancer is not fully understood. Here, we identified a novel oncogenic lncRNA termed as *lncPARP1*, which was significantly up-regulated in HCC. Increase in *lncPARP1* expression was associated with age, α-fetoprotein (AFP) levels, tumor size, recurrence, and poor prognosis of HCC patients. Loss-of-function approaches showed that knockdown of *lncPARP1* inhibited proliferation, migration, and invasion, while induced apoptosis in HCC cells. Moreover, mechanistic investigation demonstrated that PARP1 was an underlying target of *lncPARP1* in HCC. In summary, we provide the first evidence that *lncPARP1* exerts an oncogene to promote HCC development and progression, at least in part, by affecting poly (ADP-ribose) (PAR) polymerase 1 (PARP1) expression.

## Introduction

As one of the most common cancers, hepatocellular carcinoma (HCC) accounts for a large proportion of cancer-associated mortality worldwide, which causes more than 600000 deaths each year [1,2]. In spite of the remarkable improvements in comprehensive HCC treatment, such as surgery, chemotherapy, radiotherapy, and targetted therapy, the prognosis of patients with HCC remains unsatisfactory. Therefore, it is extremely urgent to reveal the molecular mechanisms in the course of HCC development, which will be greatly helpful to identify novel diagnostic and therapeutic markers for HCC patients.

Long noncoding RNAs (lncRNAs) are a class of noncoding RNAs with larger than 200 nts in length but lack the significant protein-coding capacity. Initially, lncRNA was identified as the by-product of transcription by RNA polymerase Ⅱ and without biological function [3]. However, emerging evidence demonstrated that lncRNAs play crucial roles in regulating gene expression via diverse mechanisms, including epigenetic alteration, transcriptional control, and post-transcriptional modification [4,5]. Dysregulation of lncRNAs associate with HCC initiation and progression [6,7]. For instance, lincRNA-UFC1 was overexpressed in HCC tissues, and associated with tumor size, clinical stage, and patient prognosis. LincRNA-UFC1 promoted proliferation and cell cycle progression and inhibited apoptosis through interacting with the mRNA stabilizing protein HuR and enhancing β-catenin expression [8]. Although thousands of lncRNAs have been annotated, only a few lncRNAs have been functionally characterized.

Poly (ADP-ribose) (PAR) polymerase 1 (PARP1) is a DNA-dependent ADP-ribosylation transferase, which regulates protein ADP-ribosylation and the functions of the modified proteins. PARP1 has been reported to be involved in a variety of biological processes, such as DNA damage repair, chromatin organization, chemoresistance, transcriptional control, mRNA stability, and DNA methylation [9,10]. The overexpression of PARP1 has been observed in several types of human cancers and plays important roles in carcinogenesis and cancer development [11–13]. Previous studies demonstrated that loss of some tumor suppressive miRNAs leads to PARP1 up-regulation in cancer, such as *miR-216b*, let-7, *miR-345*, and *miR-221* [14–17]. However, whether lncRNAs take part in the dysregulation of PARP1 expression in HCC remains unknown.

In our previous study, we analyzed the differential expression of lncRNAs and mRNAs between HCC and adjacent non-tumor tissues through microarray detection [18]. Here, amongst these up-regulated lncRNAs, we screened out a novel functional lncRNA (GeneSymbol: BC032899) named as *lncPARP1*. We then characterized the pathological relationship of *lncPARP1* in HCC growth and progression. Further investigation showed that *lncPARP1* functions as an oncogenic lncRNA to regulate proliferation, migration, and invasion via modulating PARP1 expression.

## Materials and methods

### Patients and tissue samples

The 70 pairs of HCC and corresponding non-tumor tissues were collected from patients with HCC who initially underwent hepatectomy without any preoperative treatment at the Zhongshan Hospital of Xiamen University from 2013 to 2016. The procedure for sample collection was approved by the ethics committee of the Zhongshan Hospital of Xiamen University, and written informed consent was obtained from all the patients.

### Ethics approval and consent to participate

All protocols dealing with the patients conformed to the ethical guidelines of the Helsinki Declaration and were approved by the Medical Ethics Committee of Hospital of Zhongshan Hospital of Xiamen University.

### Cell culture

SMMC-7721, HepG2, Huh7, SK-Hep-1, PLC/PRF/5, and Bel-7402 cells were obtained from the Cell Bank of Chinese Academy of Sciences, and MHCC-97h cells were obtained from Zhongshan Hospital of Fudan University. Cells were cultured in DMEM (HyClone) supplemented with 10% (FBS; Gibco) at 37°C. Authentication of these cell lines was performed using the GenePrint 10 System (Promega) and via comparisons with the STR database.

### RNA extraction and quantitative real-time PCR

Total RNA was isolated by TRIzol reagent (Invitrogen) as per manufacturer’s instructions. cDNA was synthesized using One-Step gDNA Removal and cDNA Synthesis Kit (Transgen, Beijing, China). Quantitative real-time PCR (qRT-PCR) was performed in the Lightcycle 96 Real-Time PCR System (Roche) using FastStart Universal SYBR Green Master (Rox) (Roche). The gene-specific primers are shown in Supplementary Table S1. ACTB was employed as an endogenous control. Comparative quantitation was determined using the 2^−ΔΔ*C*^_t_ method.

### Construction of stable cells with lncPARP1 knockdown

Three different shRNAs targetting *lncPARP1* were inserted into the pLV-shRNA-puro plasmid (Inovogen Tech, Beijing, China). The target sequences of *lncPARP1* shRNAs are shown as follows: sh1: CGGCTGACACAAGGAACTTT, sh2: CAGGAGATGGAGGAACAACA, and sh3: CGCCTCCAGATTGAACTGTCT. The stable clones expressing scramble shRNA were taken as control and the sequence was: CCTAAGGTTAAGTCGCCCTCGCTCGAGCGAGGGCGACTTAACCTTAGG. Lentiviral expressing and packaging vectors were transfected into HEK-293T cells by using TurboFect Transfection Reagent (Thermo Scientific) according to the manufacturer’s instructions. Then the medium containing the lentivirus was harvested and transduced into HCC cells in the presence of 5 μg/ml polybrene (Sigma). Stable cells were selected using puromycin for 1 week.

### Cell proliferation and colony formation assay

For cell proliferation assay, 3 × 10^3^ cells per well were seeded in 96-well culture plates. At the indicated time points, CCK-8 (Dojindo) was added to each well and incubated at 37°C for 1.5 h. The absorbance values were measured using a microplate reader (Bio–Rad) at 450 nm wave length. For colony formation assay, 3 × 10^3^ cells per well were seeded in the six-well culture plates. After 14 days’ culture, cells were fixed with 4% paraformaldehyde and stained with Crystal Violet.

### Apoptosis analysis

The apoptosis was detected by using Apoptosis Detection Kit (Dojindo) according to the manufacturer’s instructions. The data were analyzed by Kaluza software.

### Migration and invasion assay

HCC cells (3 × 10^5^) were suspended and seeded in 200 μl serum-free DMEM in the upper chamber of a 24-well Transwell migration (Corning) or invasion insert (BD Biosciences). The lower chamber was filled with DMEM containing 10% FBS. After 24 h of incubation, the cells that had traversed the membrane were fixed in 4% paraformaldehyde and then staining by Crystal Violet for 30 mins.

### Western blot

Cells or tumor tissue samples were lysed with RIPA buffer (Beyotime) containing protease inhibitors cocktail (Selleck). After centrifugation, samples were loaded on to and separated using SDS/PAGE, then transferred on to PVDF membranes (Millipore). The membranes were blocked for 1 h at room temperature and incubated with the anti-β-actin (#3700, Cell Signaling Technology), anti-PARP1 (13371-1-AP, Proteintech) and anti-Caspase 3 (#9662, Cell Signaling Technology) antibodies overnight at 4°C. After washing, the blots were incubated with goat anti-rabbit (111-035-003, Jackson) or anti-mouse (115-035-003, Jackson) HRP-conjugated secondary antibodies and visualized using the Immobilon™ HRP Substrate Peroxide Solution (Millipore).

### Xenograft assay

A xenograft mouse model was developed using 5–6-week-old male BALB/c nude mice. HCC cells (5 × 10^6^) were injected subcutaneously into the right flank of the nude mice. Tumors were detected after ~14 days, and tumor size was measured every 3 days. Tumor-bearing mice were killed 38 days after the injection, and tumors were removed for further analysis.

### Immunohistochemical

Four micrometer sections were pretreated before immunohistochemical (IHC) staining. After deparaffinization and an antigen retrieval step, endogenous peroxidase was quenched by incubation of the sections with 0.3% hydrogen peroxide for 15 min at room temperature. The sections were blocked by non-immune serum for 15 min at room temperature and then incubated with anti-PARP1 antibody (1:200, Proteintech, 13371-1-AP) or proliferating cell nuclear antigen (PCNA) antibody (1:200, Proteintech, 10205-2-AP) at 4°C overnight. After washing, the sections were incubated with biotinylated secondary antibody (KIT-5010, Maixin Biotechnology, Fuzhou, China), and then visualized using diaminobenzidine (Maixin Biotechnology, Fuzhou, China).

### Statistical analysis

All experiments were performed in triplicate. Statistical analyses were performed by using the SPSS software (version 19.0). Data are shown as mean ± S.E.M. The differences between groups were analyzed by the Student’s *t* test or chi-square test. The Kaplan–Meier method was performed for patients’ overall survival analysis. *P*<0.05 was considered statistically significant difference.

## Results

### lncPARP1 is overexpressed in HCC tissues and correlates with poor prognosis of HCC patients

**Figure 1:**
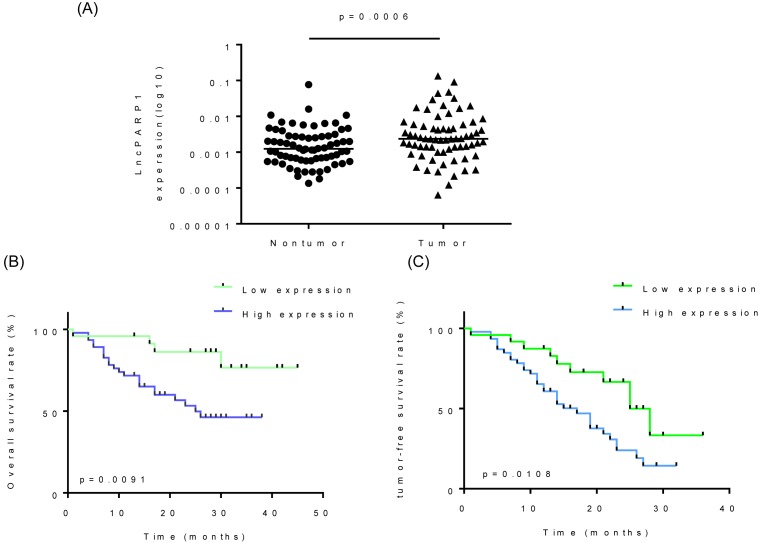
Expression of *lncPARP1* is up-regulated in HCC, which is clinically correlated with the poor prognosis of patients with HCC (**A**) Expression of *lncPARP1* in 70 pairs of HCC and corresponding non-tumor tissues was detected with qRT-PCR. (**B**,**C**) Kaplan–Meier curves and log-rank tests were used to assess the relevance of *lncPARP1* expression with (B) disease-free and (C) overall survival of patients with HCC. The patients were divided into high- and low-*lncPARP1* groups based on median of *lncPARP1* expression in HCC tissues.

First, we analyzed the differential expression of lncRNA and mRNA between HCC and adjacent non-tumor tissues through microarray detection from our previous study [18]. We focussed on the tumoral up-regulated lncRNAs nearby cancer-related protein coding genes and found an overexpressed lncRNA (GeneSymbol: BC032899) named *lncPARP1*, which was located downstream of *PARP1* gene. We then performed qRT-PCR to examine *lncPARP1* expression levels in 70 pairs of HCC and corresponding adjacent non-tumor tissue samples (Figure 1A). *lncPARP1* levels were significantly increased in HCC tissues compared with that in the non-tumor tissues. To clarify the clinical significance of *lncPARP1*, we analyzed the correlation between *lncPARP1* expression and clinicopathologic features of HCC patients. Intriguingly, *lncPARP1* up-regulation was markedly associated with elder age, higher level of serum α-fetoprotein (AFP), larger tumor size, and recurrence. No significant correlation between *lncPARP1* expression and other factors was observed, such as gender, differentiation, and invasion (Table 1). Moreover, Kaplan–Meier and log-rank test analyses suggested a significant correlation between the *lncPARP1* expression and dramatically decreased overall survival and tumor-free survival rates (Figure 1B,C). Above all, these results indicate that up-regulation of *lncPARP1* may be involved in HCC progression.

**Table 1:** The correlation between *lncPARP1* expression and clinical-pathologic features of HCC patients

Parameters	*lncPARP1* expression		*P* -values
	High	Low	
Age (years)			
<60	30	18	0.011
≥60	16	6	
Gender			
Male	39	18	0.318
Female	7	6	
AFP (ng/ml)			
<400	19	18	0.007
≥400	27	6	
Tumor size (cm)			
<3	2	5	0.029
≥3	44	19	
Differentiation			
Well moderate	37	22	0.22
Poor	9	2	
Recurrence (2 years)			
No	15	17	0.02
Yes	31	7	
Invasion			
No	19	13	0.305
Yes	27	11	

The median of *lncPARP1* expression in HCC tissues was used as cutoff.

### *lncPARP1* promotes proliferation and inhibits apoptosis in HCC cells

**Figure 2:**
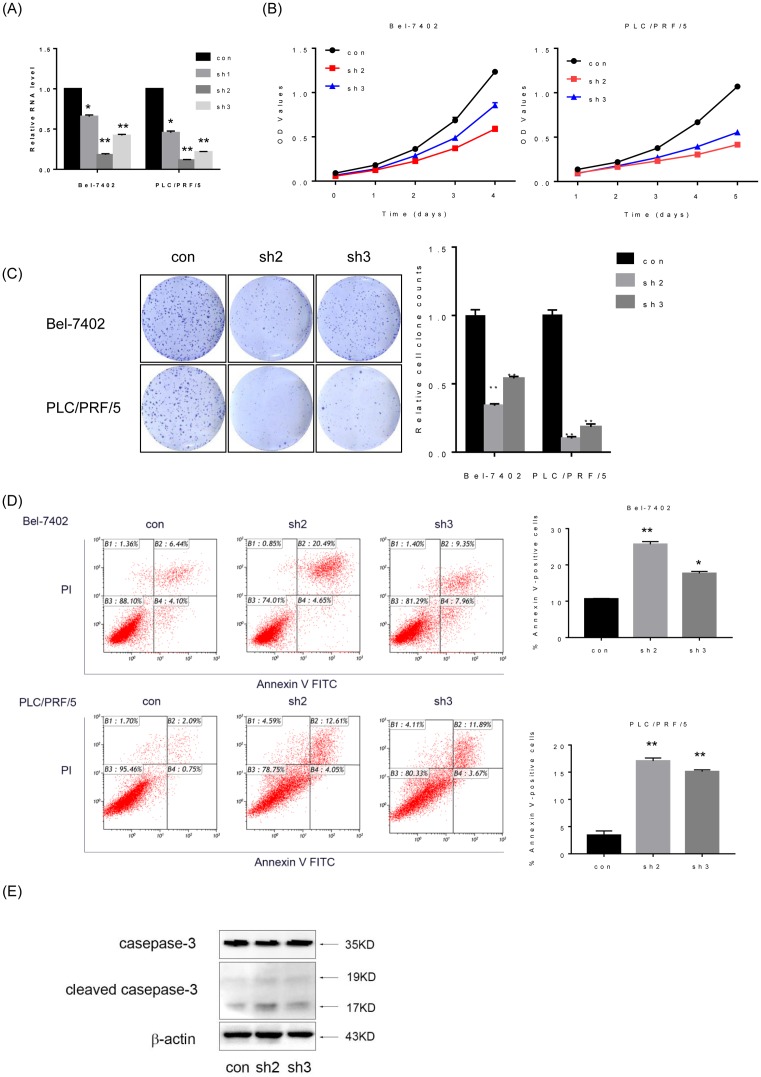
*lncPARP1* regulates HCC cell proliferation and apoptosis *in vitro* (**A**) Relative *lncPARP1* expression in Bel-7402 and PLC/PRF/5 cells transduced with lentivirus expressing control shRNA (con), *lncPARP1* shRNA1 (sh1), shRNA2 (sh2), or shRNA3 (sh3) was determined by performing qRT-PCR. (**B**) Proliferation of control and *lncPARP1*-knockdown cells was assessed by performing the CCK-8 assay. (**C**) Colony formation assays were performed using control and *lncPARP1*-knockdown cells. (**D**) *lncPARP1*-knockdown cells were treated with 5-fluorouracil (100 mg/ml) for 48 h, stained with Annexin V and propidium iodide (PI), and analyzed by flow cytometry. Annexin V-positive cells were designated as apoptotic cells. Percentage of apoptotic cells is shown. (**E**) The expression of casepase-3 and cleaved caspase-3 in Bel-7402 cells with *lncPARP1* knockdown detected by Western blot. *P<0.05, **P<0.01.

We then investigated the functional roles of *lncPARP1* in HCC progression. To choose the HCC cell lines used for loss-of-function assays, we detected the *lncPARP1* expression in seven different HCC cell lines, including Bel-7402, SMMC-7721, MHCC-97h, HepG2, Huh7, PLC/PRF/5, and SK-Hep-1 cells. Amongst these HCC cells, Bel-7402 and PLC/PRF/5 cells expressed the highest level of *lncPARP1* (Supplementary Figure S1). Therefore, Bel-7402 and PLC/PRF/5 cells were selected for knockdown of *lncPARP1*. To avoid off-target effects, we designed three independent shRNAs against *lncPARP1*, and the two most efficient (sh2 and sh3) were selected for further experiments (Figure 2A). To analyze the effects of *lncPARP1* silence on HCC cell proliferation, we performed CCK-8 assay and found that knockdown of *lncPARP1* remarkably inhibited proliferation of both Bel-7402 and PLC/PRF/5 cells, compared with that of control cells (Figure 2B). Similarly, *lncPARP1* inhibition by shRNAs significantly suppressed the colony-forming ability of HCC cells (Figure 2C).

To further determine mechanisms underlying the *lncPARP1*-mediated cell proliferation, we checked the influence of *lncPARP1* knockdown on cell cycle distribution by performing flow cytometry. However, the results illustrated that *lncPARP1* silence did not affect cell cycle progression, suggested that *lncPARP1* did not modulate cell cycle (Supplementary Figure S2). Given that *lncPARP1* functions as an oncogenic lncRNA in HCC cells, we speculated that *lncPARP1* may be critical for cell survival and apoptosis. To verify this hypothesis, we analyzed the apoptosis by performing flow cytometry in HCC cells with Annexin V and propidium iodide (PI) staining. As shown in Figure 2D, lncPARP1-silencing Bel-7402 and PLC/PRF/5 cells showed a significantly higher percentage of annexin V-positive cells than did control cells. Consistent with this result, the well-known apoptosis protein marker, cleaved Caspase 3, were markedly increased by *lncPARP1* knockdown (Figure 2E).

### Knockdown of *lncPARP1* represses migration and invasion in HCC cells

**Figure 3:**
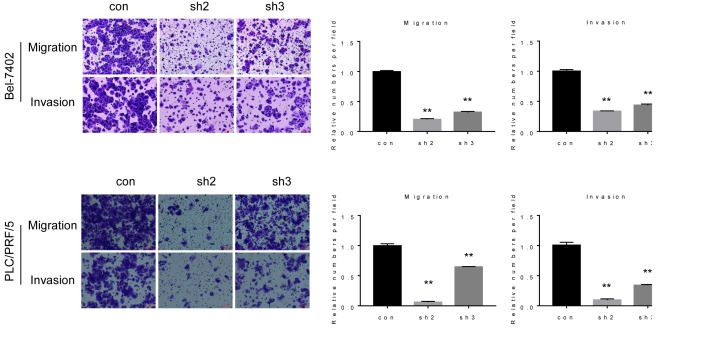
lncPARP1 enhances HCC migration and invasion Left, representative images of the migration and invasion of Bel-7402 and PLC/PRF/5 cells expressing control and lncPARP1 shRNAs. Right, statistical results obtained from three independent experiments. **P<0.01.

We next investigated the role of *lncPARP1* in HCC cell migration and invasion. Cell motility was measured by migration assay. The results showed that depletion of *lncPARP1* suppressed the migratory capacity of both Bel-7402 and PLC/PRF/5 cells (Figure 3). We then used Matrigel-coated Transwell experiments to examine HCC cell invasion, and the result revealed that the number of Bel-7402 and PLC/PRF/5 cells invading through membrane was significantly reduced after *lncPARP1* silencing (Figure 3).

### *lncPARP1* positively modulate PARP1 expression

**Figure 4:**
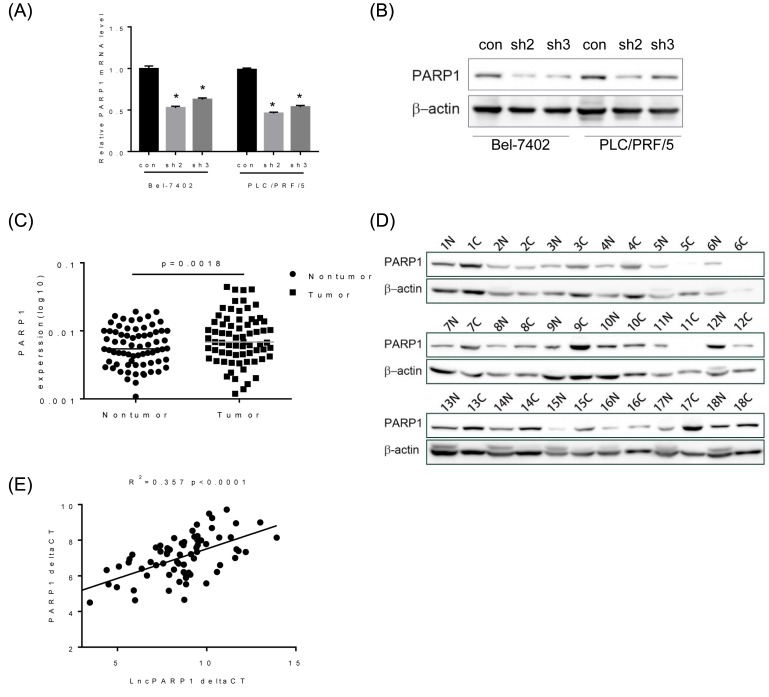
The correlation between *PARP1* and *lncPARP1* expression in HCC (**A**,**B**) *PARP1* mRNA (A) and protein expression (B) in control and *lncPARP1* knockdown HCC cells was analyzed by performing qRT-PCR and Western blot, respectively. (**C**) *PARP1* mRNA expression in 70 pairs of HCC and corresponding non-tumor tissues was determined by performing qRT-PCR. (**D**) PARP1 protein expression in 18 pairs of HCC and peritumor tissues were analyzed by Western blot. (**E**) The correlation between *PARP1* and *lncPARP1* in HCC tissue samples. *P<0.05.

lncRNAs act in *cis* or *trans* to regulate the expression of neighboring protein-coding genes [19]. *LncPARP1* is an intergenic lncRNA, which was localized downstream of *PARP1* gene. Therefore, we speculated that *lncPARP1* could affect PARP1 expression. To test this hypothesis, we detected the mRNA and protein levels of PARP1 in Bel-7420 and PLC/PRF/5 expressing *lncPARP1* shRNAs. Interestingly, depletion of *lncPARP1* significantly repressed both mRNA and protein levels of PARP1 (Figure 4A,B). We further clarified the pathological relationship between *lncPARP1* and *PARP1* expression. We examined *PARP1* mRNA expression in 70 pairs of HCC and paratumor tissues by qRT-PCR. The results showed that *PARP1* mRNA expression was much higher in HCC tissues than that in adjacent non-tumor tissues (Figure 4C). The result of Western blot also demonstrated an increase in *PARP1* in HCC tissue samples (Figure 4D). Moreover, a positive correlation was observed between *lncPARP1* and *PARP1* expression levels in HCC tissues (*R^2^*= 0.357, *P*<0.0001, Figure 4E), supporting that PARP1 expression is regulated by *lncPARP1*.

### Deletion of *lncPARP1* suppresses HCC growth *in vivo*

**Figure 5:**
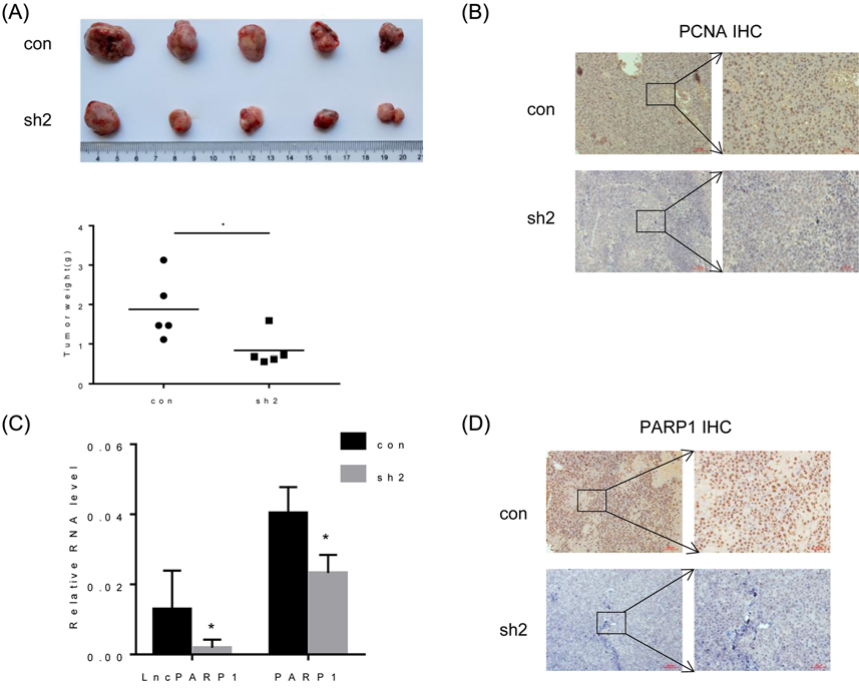
Effects of lncPARP1 knockdown on HCC growth *in vivo* (**A**) Upper, representative images of tumors formed in nude mice subcutaneously injected with control and *lncPARP1*-knockdown PLC/PRF/5 cells; Below, tumor weight measured 6 weeks after injecting. (**B**) Proliferation marker PCNA expression in tumor tissues formed by control and *lncPARP1*-knockdown PLC/PRF/5 cells was examined by IHC. (**C**) *lncPARP1* and PARP1 RNA expression of in tumor tissues formed by control and *lncPARP1*-knockdown PLC/PRF/5 cells was analyzed by qRT-PCR. (**D**) The PARP1 protein expression in tumor tissues formed by control and *lncPARP1*-knockdown PLC/PRF/5 cells was examined by performing IHC staining. *P<0.05.

To further confirm the function of *lncPARP1* during process of HCC growth, we used a xenograft mouse model that was generated by subcutaneously injecting control and *lncPARP1*-knockdown PLC/PRF/5 cells into nude mice. Xenografted tumors derived from *lncPARP1*-knockdown PLC/PRF/5 cells had smaller volumes and expressed lower level of PCNA than tumors derived from control cells (Figure 5A,B). Consistent with *in vitro* observation, both mRNA and protein levels of *PARP1* in *lncPARP1* knockdown tumors were much lower than that in control tumors (Figure 5C,D). Collectively, our findings suggest that *lncPARP1* promotes tumor growth through up-regulation of *PARP1 in vivo*.

## Discussion

*LncRNAs* have emerged as critical regulators in HCC tumorigenesis and progression. Nevertheless, the biological functions and underlying mechanistic details for most lncRNAs in HCC is still elusive. The key finding of our current study is that a novel lncRNA *lncPARP1* is significantly up-regulated in HCC tissues. Increase in *lncPARP1* expression is closely associated with age, AFP level, tumor size, and recurrence of HCC patients. High-level *lncPARP1* expression is a potential predictor for poor tumor-free and overall survival of HCC patients. We then determined the relationship between *lncPARP1* expression and malignant phenotypes of HCC cells using loss-of-function approaches. Knockdown of *lncPARP1* inhibited proliferation, migration, and invasion, while induced apoptosis in HCC cells. This is the first time to report the clinical and functional significance of *lncPARP1* expression contributing to HCC tumorigenesis and progression.

PARP1 is the major member of Parps family to promote PAR chain formation through DNA-dependent modulation. PARP1-mediated poly(ADP-ribosyl)ation (PARylation) is a post-translational modification, which regulates protein–protein interaction and transcription [20,21]. Abnormally expressed PARP1 is involved in carcinogenesis. Specifically, PARP1 is crucial in eliciting the protection against genotoxic effects via regulating the DNA repair mechanism, PARP1 interacts with P53 and promotes its PARylation to inhibit binding affinity of p53 to target genes during apoptosis program [22], implying that *lncPARP1* may regulate the DNA repair in response to DNA damage through *PARP1*-p53 signaling. *PARP1* also plays important role in mesenchymal–epithelial transition (EMT) which promotes tumor metastasis. PARP1 associates with EMT-related transcription factor Snail. PARP1 mediated PARylation affects the stability of Snail to modulate EMT process [23]. Thus, these studies indicate a crucial role of PARP1 in cancers development. Recently, it has been reported that some lncRNAs regulate cancer progression through interaction with PARP1. For example, overexpression of lncRNA *FOXD3-AS1* induces neuronal differentiation and decreases the aggressiveness of neuroblastoma cells through interacting with *PARP1* and inhibiting the activation of CCCTC-binding factor (CTCF) [24]. However, to the best of our knowledge, whether lncRNAs take part in the increase in *PARP1* expression in cancer remains unknown. Our present study showed that knockdown of *lncPARP1* significantly suppressed both mRNA and protein levels of PARP1 expression *in vitro* and *in vivo*. Moreover, a positive correlation between *lncPARP1* and *PARP1* expression in HCC tissues was observed, suggesting that *PARP1* was a *bona fide* target gene of *lncPARP1*. Unfortunately, we did not reveal the exact mechanism of *PARP1* expression induced by *lncPARP1*. Previous studies demonstrated that intergenic lncRNAs exert functions through interaction with epigenetic modifier to regulate nearby protein-coding gene expression [25]. For example, lncRNA-HEIH was associated with EZH2 and repressed EZH2 target genes, such as *p16, p21*, and *p57* [26]. LncTCF7 activated TCF7 expression to promote self-renewal of HCC stem cells through interaction with the SWI/SNF complex [25]. Therefore, we suspect that *lncPARP1* modulates *PARP1* expression in such a manner, which needs further investigation.

## Conclusion

In conclusion, we characterized a new functional lncRNA *lncPARP1* that regulates the expression of PARP1 and is a novel molecule involved in the progression of HCC. Our findings suggest that *lncPARP1* might be used as a promising prognostic and therapeutic marker of HCC.

## Supporting information

**supplementary Figure 1:** LncPARP1 expression in the immortalized normal liver cells (LO2cells) and seven HCC cell lines was analyzed by performing qPCR. LO2 cells were used as controls. *p < 0.05(Student’s t test).

**supplementary Figure 2:** Cell cycle of HCC cells was determined by flow cytometry, which showed no significant difference between contorl cells and LncPARP1 knockdown cells.

**Supplemental Table 1:** The gene-specific primers of LncPARP1, PARP1 and GAPDH (internal reference) for performing qPCR (Upper) and three independent RNA interference sequences of LncPARP1.

## Abbreviations

ACTB: beta-actin
AFP: α-fetoprotein
CCK-8: cell counting kit-8
DMEM: Dulbecco’s Modified Eagle’s medium
EMT: mesenchymal–epithelial transition
EZH2: Enhancer of zeste homolog 2
HCC: hepatocellular carcinoma
HRP: Horseradish peroxidase
lincRNA: long intergenic noncoding RNA
lncRNA: long noncoding RNA
PAR: poly (ADP-ribose)
PARP1: PAR polymerase 1
PCNA: proliferating cell nuclear antigen
qRT-PCR: quantitative real-time PCR
RIPA: Radio Immunoprecipitation Assay
SWI/SNF: SWItch/Sucrose Non-Fermentable
UFC1: ubiquitin-fold modifier conjugating enzyme 1
