# Supplementary material for: The long noncoding RNA lncPARP1 contributes to progression of hepatocellular carcinoma through up-regulation of PARP1
Source: Biosci Rep. 2018 Jun 21;38(3):BSR20180703. doi: 10.1042/BSR20180703 (PMC6013699; doi:10.1042/BSR20180703)
Supplement: Supplementary file 1 [file bsr20180703_Supp1.pdf]

Supplemental Figure 1. LncPARP1 expression in the immortalized normal liver cells (LO2cells) and seven HCC cell lines was analyzed by performing qPCR. LO2 cells were used as controls. *p < 0.05(Student’s t test).

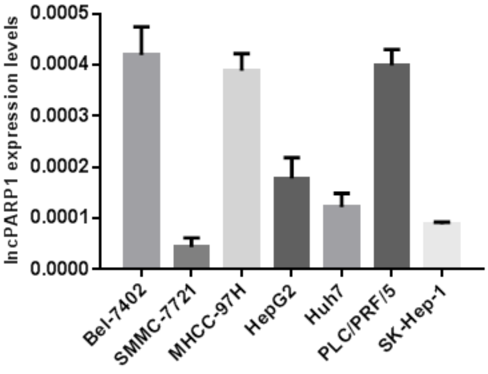

Supplemental Figure 2. Cell cycle of HCC cells was determined by flow cytometry, which showed no significant difference between contorl cells and LncPARP1 knockdown cells.

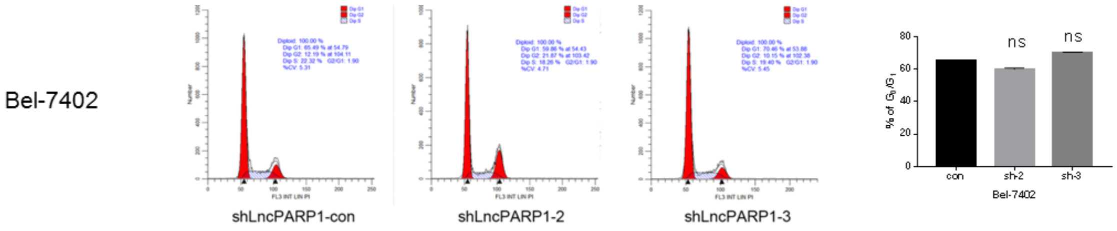

Supplemental Table 1: The gene-specific primers of LncPARP1, PARP1 and GAPDH (internal reference) for performing qPCR (Upper) and three independent RNA interference sequences of LncPARP1.

qRT-PCR	Primer name	Forward	Reverse
	LncPARP1	ATACCCTAGGAGATGGAGGAAC	GGCAACAGTGGCTTGAGATA
	PARP1	GCCGAGATCATCAGGAAGTATG	ATTCGCCTTCACGCTCTATC
	GAPDH	CTTTGGTATCGTGGAAGGACTC	AGTAGAGGCAGGGATGATGT

shRNA	LncPARP1-1	GATCCGGCTGACACAAGGAACTTTTTCAAGAGAAAAGTTCCTTGTGTCAGCCTTTTTTG	AATTCAAAAAAGGCTGACACAAGGAACTTTTCTCTTGAAAAAGTTCCTTGTGTCAGCCG
	LncPARP1-2	GATCCAGGAGATGGAGGAACAACATTCAAGAGATGTTGTTCCTCCATCTCCTTTTTTTG	AATTCAAAAAAAGGAGATGGAGGAACAACATCTCTTGAATGTTGTTCCTCCATCTCCTG
	LncPARP1-3	GATCCGCCTCCAGATTGAACTGTCTTTCAAGAGAAGACAGTTCAATCTGGAGGTTTTTTG	AATTCAAAAAACCTCCAGATTGAACTGTCTTCTCTTGAAAGACAGTTCAATCTGGAGGCG
